# Role of Flavonoids as Epigenetic Modulators in Cancer Prevention and Therapy

**DOI:** 10.3389/fgene.2021.758733

**Published:** 2021-11-09

**Authors:** Nishat Fatima, Syed Shabihe Raza Baqri, Atrayee Bhattacharya, Nii Koney-Kwaku Koney, Kazim Husain, Ata Abbas, Rais A. Ansari

**Affiliations:** ^1^ Department of Chemistry, Shia Postgraduate College, Lucknow, India; ^2^ Department of Zoology, Shia Postgraduate College, Lucknow, India; ^3^ Department of Medical Oncology, Dana-Farber Cancer Institute, Harvard Medical School, Boston, MA, United States; ^4^ Department of Anatomy, University of Ghana Medical School, College of Health Sciences, University of Ghana, Accra, Ghana; ^5^ Department of Molecular Medicine, University of South Florida, Tampa, FL, United States; ^6^ Division of Hematology and Oncology, Department of Medicine, Case Western Reserve University, Cleveland, OH, United States; ^7^ Case Comprehensive Cancer Center, Case Western Reserve University School of Medicine, Cleveland, OH, United States; ^8^ Department of Pharmaceutical Sciences, Nova Southeastern University, Fort Lauderdale, FL, United States

**Keywords:** cancer, flavonoids, epigenetics, DNA methylation, histone modifications, non-coding RNAs

## Abstract

Epigenetic regulation involves reversible changes in histones and DNA modifications that can be inherited without any changes in the DNA sequence. Dysregulation of normal epigenetic processes can lead to aberrant gene expression as observed in many diseases, notably cancer. Recent insights into the mechanisms of DNA methylation, histone modifications, and non-coding RNAs involved in altered gene expression profiles of tumor cells have caused a paradigm shift in the diagnostic and therapeutic approaches towards cancer. There has been a surge in search for compounds that could modulate the altered epigenetic landscape of tumor cells, and to exploit their therapeutic potential against cancers. Flavonoids are naturally occurring phenol compounds which are abundantly found among phytochemicals and have potentials to modulate epigenetic processes. Knowledge of the precise flavonoid-mediated epigenetic alterations is needed for the development of epigenetics drugs and combinatorial therapeutic approaches against cancers. This review is aimed to comprehensively explore the epigenetic modulations of flavonoids and their anti-tumor activities.

## Introduction

**FIGURE 1:**
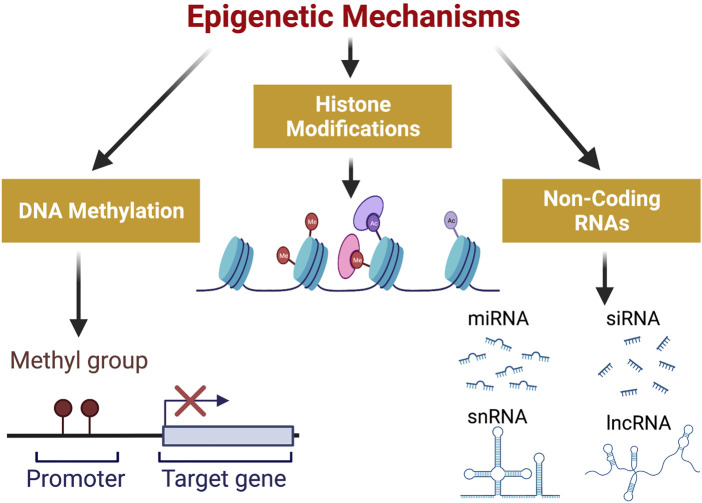
Epigenetic Mechanisms involved in gene expression: DNA methylation, Histone Modifications and Non-Coding RNAs constitute the three mechanisms involved in epigenetic regulation of gene expression. Methylation of DNA occurs at gene promoters and hypermethylation represses gene expression. Histone modifications include acetylation and methylation of histones which can either activate or repress gene expression. Non-coding RNAs which consist of short chain non-coding RNAs ( <200 nt) and long non-coding RNAs ( >200 nt) also play a significant role in regulation of gene expression. (Created with BioRender.com). Ac, acetylated histone; Me, methylated histone; miRNA, micro RNA; siRNA, small interfering RNA; snRNA, small nuclear RNA; lncRNA, long non-coding RNA.

Epigenetics can be defined as the study of heritable changes in gene expression without involving any changes in the DNA sequence (Weinhold, 2006). Epigenetic changes are established during early differentiation of cells and stably inherited through multiple cell divisions resulting in distinct cellular phenotypes without any changes in the underlying DNA sequence (Cheedipudi et al., 2014). The process of epigenetic regulation of gene expression involves chromatin remodeling mediated by events like DNA methylation, histone modifications and effects of non-coding RNAs as shown in Figure 1. These epigenetic changes are essential for normal development of cells, but their deregulation can lead to certain disease states including cancer (Egger et al., 2004).

There is a consensus among biologists that cancer can be considered as a genetic inevitability. DNA mutations accumulate in the cells as we age. The mutations can be either spontaneous due to errors of DNA replication or may be caused by physical or chemical mutagens. Hence, 90–95% of cancers involve mutations due to environmental or lifestyle factors and in the remaining 10% of cases, the genetic cause of cancer is hereditary (Anand et al., 2008). Interestingly, many of the gene defects in cancers are not due to changes in sequences but are a result of epigenetic changes that affect the expression profile of these genes (Taby and Issa, 2010; Cavalli and Heard, 2019). Recent advances in the field of epigenetics have highlighted global epigenetic abnormalities in cancer cells which can occur during the early stages of tumor development. Due to their reversible nature and early occurrence in the process of malignant transformation of normal cells, epigenetic modifications can serve as novel drug targets for the treatment of cancer (Dawson and Kouzarides, 2012; Bennett and Licht, 2018).

Even though, in 2020, the global estimate of new cancer cases was 19.3 million which resulted in almost 10 million deaths, cancer related deaths have been declining since 1991 leading to a decrease of 31% in 2018 (Siegel et al., 2021). This improvement in patient’s survival rate is a result of advancement in cancer treatments. Cancer treatments usually involve chemotherapeutic agents, radiotherapy, and immunotherapy, and these treatments have shown a lot of promise (Yu et al., 2019). Epigenetic modulators (e.g., histone deacetylases inhibitors) have been used to treat aberrant epigenetic modifications in cancer along with some novel approaches such as chimeric antigen receptor-engineered T cells (CAR-T cells) which have proven very efficient in treating malignancies of B-cells (Wang et al., 2020b). However, most of the cancer therapies including immunotherapy are associated with numerous side effects (Lacouture and Sibaud, 2018).

Thus, considering the adverse effects of radiation and chemotherapeutic agents on patients, there is an increased quest for alternative therapies without extreme side effects. Phytochemicals fit best in this criterion and are hence explored for their anticancer properties. Anticarcinogenic factors in plant-based foods are known to inhibit cancer by a variety of mechanisms ranging from antioxidant effects to immunomodulatory properties. Interestingly, it has been reported that phytochemicals can modulate epigenetic processes through DNA methyltransferases (DNMTs) and histone deacetylases (HDACs) (Shukla et al., 2014). Flavonoids which constitute an important class of phytochemicals are implicated in modulation of gene expression patterns underlying cancer (Busch et al., 2015). In this review we first discuss the role of epigenetic aberrations in cancer and then present an overview of the usefulness of flavonoids as epigenetic modulators having chemotherapeutic and chemo-preventive properties.

### Cancer Epigenetics

In eukaryotic cells, DNA is wrapped around a core of histone proteins to form nucleosomes which may wrap over themselves to adopt a condensed state temporarily or permanently. The degree to which a gene is expressed in each cell is controlled by a range of gene regulatory mechanisms, most of which interfere with chromatin condensation. The condensation of chromatin involves a compact packing of nucleosomes which renders the genes hetero-chromatinzed and hence inactive. On the contrary, decondensation of chromatin opens the nucleosomes and increased expression of genes. The transformation of normal cells into cancer cells involves epigenetic alterations and in most cases is preceded by genetic mutations (Brower, 2011). Amongst the key mechanisms involved in epigenetic regulation are DNA modifications (e.g., methylation), histone modifications (e.g., deacetylation, phosphorylation, and ubiquitination, etc.), nucleosome positioning, and non-coding RNAs (e.g., miRNA, siRNA, lncRNA, etc.) (Figure 1). These mechanisms have a significant impact on cellular homeostasis (Zhang et al., 2020).

### DNA Methylation

The methylation or hydroxymethylation of DNA is an important epigenetic mechanism of gene regulation in cells (Gao and Das, 2014) and DNA methylation patterns contribute in establishing epigenetic memory (Bird, 2002). The methylation of DNA is usually negatively correlated with gene expression, but it also depends on the location of methylated bases in relation to coding regions of the genes being regulated (Blake et al., 2020). Cytosine methylation most commonly occurs at CpG sites which are widely distributed through the genome (Jones, 2012). The specific regions of CpG sites are designated as CpG islands when they have a length of more than 200 bp, a GC content of more than 55% and a >60% observed-to-expected CpG ratio (Takai and Jones, 2002). CpG islands are particularly abundant at promoter region of the gene, and are present in around 70% of human promoters (Deaton and Bird, 2011). When CpG sites are present in the promoter or enhancer regions of genes their methylation represses gene expression whereas it induces transcriptional activity if the CpG sites are present in the coding regions of genes (Greenberg and Bourc’his, 2019). The pattern of CpG methylation in cancer cells is different to that of normal cells (Esteller, 2007). In normal cells, whereas the CpG islands preceding promoters are unmethylated allowing active transcription while other CpG sites in the genome remain methylated. In cancer cells, the CpG dinucleotides have up to 50% less methylation than normal cells and CpG islands are also generally hypomethylated (Herman and Baylin, 2003). In general, promoters of tumor suppressor genes become hypermethylated thereby inhibiting their expression while those of oncogenes get hypomethylated and activated in cancer. Genes involved in DNA repair, cell cycle, migration and apoptosis are dysregulated due to aberrant DNA methylation in cancer cells (Cheung et al., 2009). This process of *de novo* DNA methylation is carried out by DNMTs and include three isoenzymes: DNMT1, DNMT3a and DNMT3b.

### Histone Modifications

Histones are positively charged proteins that play a role in condensing and packaging the DNA into chromatin inside the nucleus. Open chromatin structure (euchromatin) is associated with transcriptional activation and closed chromatin structure (heterochromatin) is associated with repression of transcription. Histone modifications especially acetylation, phosphorylation, and methylation regulate structural changes in the chromatin influencing gene expression (Karlic et al., 2010; Bannister and Kouzarides, 2011).

Histone acetylation is defined as the addition of an acetyl group to lysine residues present at the histone tail by histone acetyl transferases (HATs). This modification weakens the DNA-histone interaction eventually leading to decondensation of chromatin and increased gene expression. On the contrary, HDACs constitute an important class of enzymes and are responsible for deacetylation of *e*-amino groups of lysine residues leading to condensation of chromatin and decreased gene expression. (Milazzo et al., 2020). The pattern of histone acetylation has been found to be remarkably different between normal and cancerous cells (Di Cerbo and Schneider, 2013). Histone H4 exhibits a decrease in monoacetylation of Lys20 and trimethylation of Lys16 in malignant cells (Fraga et al., 2005). Besides, decreased acetylation of histones H3 and H4 has also been observed in cancer progression (Audia and Campbell, 2016).

Histone methylation is another epigenetic modification that plays a role in regulating gene expression in cancer. These modifications are catalyzed by Histone Methyltransferases (HMTs) and Histone Demethylases (HDMs) of the specific amino acids in histones. Cancer specific gene transcription profiles are often related to the regulation of histone methylation (Varier and Timmers, 2011). For instance, there is a cancer-associated decrease in trimethylation on Lysine 4 of histone H3 (H3K4me3) along with a simultaneous increase of monomethylation on Lysine 9 of histone H3 that affects gene expression (Richon et al., 2000).

Phosphorylation is another histone posttranslational modification mediated by cell-cycle related kinases (Rossetto et al., 2014). The phosphorylation of serine at the C-terminus of a DNA double-strand break marker H2A histone family member X (H2AX), eventually contributes to genomic instability leading to cancer (Bonner et al., 2008).

### Non-Coding RNAs

The next important epigenetic mechanism controlling cell function involves non-coding RNAs which are being shown to regulate gene expression to a great extent (Kurokawa et al., 2014; Statello et al., 2020). Small non-coding RNAs composed of 18–25 nucleotides are called MicroRNAs (miRNAs) which usually interacts with the 3’ region of the mRNA affecting mRNA stability and translation. One miRNA can regulate the expression of several genes or multiple miRNAs can affect the expression of the same gene (Mohr and Mott, 2015). Transcriptional activity of up to 60% of mammalian protein coding genes has been found to be controlled by miRNAs (Friedman et al., 2009). Several miRNAs have been associated with the regulation of oncogenes and tumor suppressor genes thereby having a role in cancer (Peng and Croce, 2016). The most common cancer associated miRNAs (onco-miRNAs) that are promising candidates for cancer treatment are let-7, miR-15, and miR-16 (Esquela-Kerscher and Slack, 2006). Also, miR-125b1 has been shown to act as a tumor suppressor as its decrease is associated with ovarian and prostate cancers (Soto-Reyes et al., 2012). The roles of micro RNAs in cancer progression are contributing to a great bulk of emerging knowledge about cancer (Ali Syeda et al., 2020). Events like genetic alterations, promoter hypermethylation, or other epigenetic modifications regulate the expression of miRNAs and promotes cellular transformation and cancer progression (Baer et al., 2013; Liu et al., 2013; Cammaerts et al., 2015).

Long non-coding RNAs (lncRNAs) are polyadenylated RNAs with a length of more than 200 nucleotides that can bind to DNA, RNA, and proteins. Epigenetic modulation is the most common method of lncRNAs based regulation of gene expression and is often associated with gene repression. Studies have reported that lncRNAs can function as oncogenes or tumor suppressors through a wide range of activities, including interaction with Polycomb Repressive Compex (PRC) regulating transcript stability, processing and translation; interaction with miRNAs, gene enhancers and repressors; and interaction with transcription factors affecting transcript production and transport (Marín-Béjar et al., 2013; Vance and Ponting, 2014; Marchese et al., 2017; Zhang et al., 2019b; Hou et al., 2019). LncRNAs like MALAT1 and HOTAIR which are associated with metastasis are over expressed in cancer and MEG3 and PTENP1 which inhibit cell proliferation and migration are downregulated in cancer (Huarte, 2015).

### Flavonoids as Epigenetic Modulators

Flavonoids belong to an important class of natural low-molecular-weight polyphenolic compounds having basic benzo- γ -pyrone structure (Panche et al., 2016). These plant secondary metabolites are widely found in various vegetables, fruits, cereal, nuts and specially in certain beverages (tea, coffee). Flavonoids are linked with a wide spectrum of health-promoting goods and are an important constituent in a range of pharmaceutical, nutraceutical, cosmetic and medicinal applications. Flavonoids have several subgroups based on their chemical structures, which comprise flavan-3-ols, flavonols, flavones, flavanones, isoflavones, and anthocyanidins (Kumar and Pandey, 2013). Furthermore, flavonoids possess a wide array of beneficial pharmacological activities including antiviral, hepatoprotective, antibacterial, analgesic, cytostatic, antiallergic, anti-oestrogenic, oestrogenic and apoptotic (Kumar and Pandey, 2013). These assorted pharmacological activities of flavonoids have been accredited to several molecular mechanisms including direct and indirect antioxidant task, modulation of the activities of phase I and II detoxification enzymes, inhibition of protein kinases, modulation of gene transcription, consequence on cell cycle and epigenetic mechanisms (Pietta, 2000; Woo et al., 2005; Kumar and Pandey, 2013; Miron et al., 2017; Jucá et al., 2018; Khan et al., 2021). There are recent reports indicating that flavonoids may reinstate the standard epigenetic marks which are changed during carcinogenesis (Carlos-Reyes et al., 2019; Jiang et al., 2021; Khan et al., 2021). Generally, these photochemical agents block the progress of tumors by targeting key signaling transducers resulting in the reinstatement of tumor suppressor genes, and blocking of oncogenes expression. These alterations and resulting anti-tumor activities often come from epigenetic modulatory activities of flavonoids by altering epigenetic enzymes such as DNMTs, HDACs and HATs (Pandey et al., 2012; Abbas et al., 2013; Abbas et al., 2016; Jiang et al., 2021; Khan et al., 2021). It was also documented that flavonoids are proficient in modulating of miRNA and lncRNA expression that is changed during ailments (Izzo et al., 2020).

**FIGURE 2:**
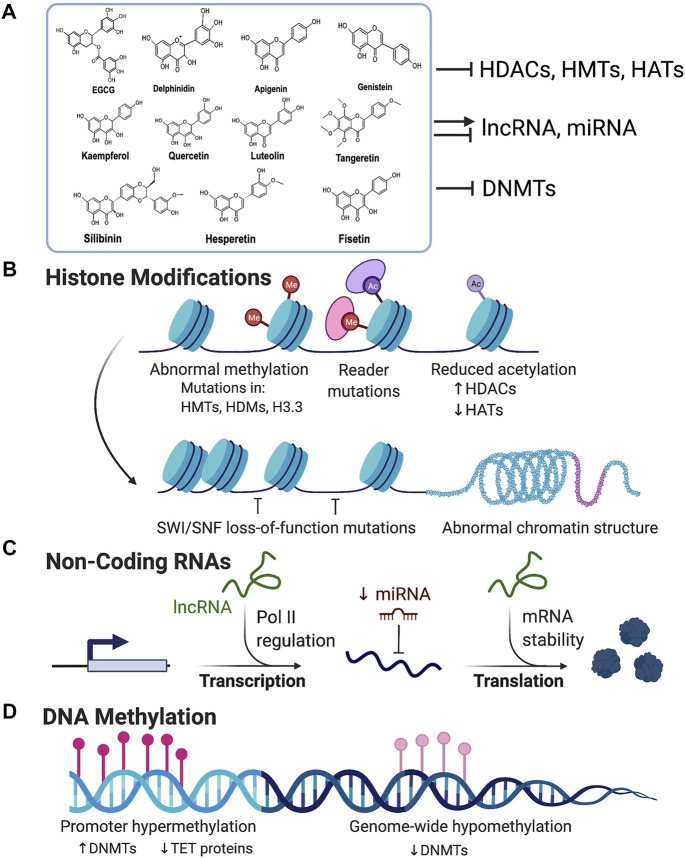
Flavonoids as epigenetic modulators in cancer: **(A)** Flavonoids are polyhydroxy compounds found in various plants and generally consist of two phenyl rings and a heterocyclic ring. Flavonoids are reported to exhibit inhibitory activity for HDACs, HMTs, HATs, and DNMTs. They can also either inhibit or activate miRNA and lncRNA. **(B)** Illustration showing various mechanisms of histone modifications associated with carcinogenesis. Flavonoids can block the aberrant expression of HMTs, HATs, and HDACs, and activate tumor suppressor genes and block the expression of oncogenes. **(C)** Flavonoids can either activate or repress non-coding RNAs which regulates chromatin structure and aberrant gene expression in cancer. **(D)** Promoter hypermethylation and genome-wide hypomethylation are associated with cancer. Flavonoids can inhibit DNA Methyl Transferases (DNMTs) and thus prevent hypermethylation of gene promoters like tumor suppressor genes thereby reactivating their expression. (Created with BioRender.com).

Despite the lifesaving role of chemotherapeutic agents in treating cancer, a disadvantage of these drugs is their potential cytotoxic effects on normal cells. Thus, there is a need for better substitutes without undesirable side-effects. In this regard, flavonoids show promising results as many of the anticarcinogenic flavonoids have relatively less toxicity towards normal cells (Galati and O'Brien, 2004); however, more in-depth toxicity studies are needed to evaluate their safety and side-effects. It is also documented that on normal cells they may act as pro-oxidants that generate free radicals, mutagens and act as inhibitors of key enzymes involved in hormone metabolism when consumed at higher doses (Skibola and Smith, 2000). Nonetheless, flavonoids having epigenetic modifying potential can be an attractive choice for potential cancer therapies, including combinatorial therapy (Figure 2; Table 1).

**TABLE 1:** Epigenetic modulations by some potent flavonoids (↑ increase, ↓ decrease).

Phytochemical	Dose	Epigenic modulation	Gene targets	Cancer type	Cancer cell line	Biological approach	Biological effect	References
EGCG (Flavanol)	0–100 μM	↓ DNMT	*SCUBE2*, vimentin	Breast cancer	MCF-7 and MDA-MB-231	*in vitro*	↓ Cell viability,	Sheng et al. (2019)
							↓ Cell migration	
							↓ Invasion	
	5–50 M	↓ DNMT	p16, *RARB*, *MGMT*, *hMLH1*	Esophageal, prostate and colon cancer	KYSE 510, PC3, HT-29, KYSE 150	*in vitro/ in silico*	↑ Apoptosis	Fang et al. (2003)
		↓ DNMT	*GAS1*, *TIMP4*, *ICAM1,* and *WISP2*	Non-small-cell lung cancer	NSCLC, A549/DDP	*in vitro/in vivo*	↓ Cell proliferation	Zhang et al. (2015)
		↓ HDAC					↑ Apoptosis	
	0.5% in diet	↓ DNMT1	*Cdh13*, *Prdm2*, and *Runx3*	Lung cancer	-	*in vivo*	p-AKT, and γ-H2AX inductions	Jin et al. (2015)
	20 µM	↓ HDAC	*MMP-2/MMP-9, EZH2,* and *TIMP*-3	Prostate cancer	DUPRO and LNCaP cells	*in vitro/clinical trial*	↓ Cell invasion and migration	Deb et al. (2019)
	5–20 µM	↓ HDAC1, 2, 3 and 8	p53	Prostate cancer	LNCaP	*in vitro*	↑ Apoptosis	Thakur et al. (2012)
							Cell cycle arrest	
	50 µM	↓ HAT	p300	Prostate cancer	LNCaP, PC3	*in vitro*	↑ Apoptosis	Lee et al. (2012)
	1–10 μg/ml	↓ DNMT	*GSTP1*, *S100P*	Prostate cancer	LNCaP	*in vitro*	↑ Apoptosis growth arrest	Pandey et al. (2010)
	0.2–400 μM	↓ mRNA	*Ccna2*, *Ccnb1*, *Ccnd1*, *Ccne1*, *E2f1*, *Dr5*, *p21*, *Cd24*, *Cd133*, *Abcg2*, *Eed*, *Ezh2*, and *Suz12*	Biliary tract cancer	CCSW-1, EGI-1, GBC, MzChA-1, MzChA-2, TFK-1, BDC and SkChA-1	*in vitro*	↑ Apoptosis	Mayr et al. (2015)
							↓Cell viability	
							Cell cycle arrest	
							↑ caspase activity	
	25 µM	↓ DNMT	*RARβ*, *CDH1*, *DAPK1*	Cervical cancer	HeLa	*in vitro/in silico*	↑ Apoptosis	Khan et al. (2015)
		↓ HDAC						
	-	↓ DNMT1, ↓ HDAC1, ↓ HDAC2	p27, *CAF*, C/*EBPα*, C/*EBPε, EZH2*, *SUZ12*, and *EED*	Promyelocytic leukemia	-	*in vitro*	↑ Apoptosis	Borutinskaitė et al. (2018)
							Cell cycle arrest	
		↓ HDAC	p53, p21	Lung cancer	PC-9	*in vitro*	↑ Apoptosis	Oya et al. (2017)
Quercetin (Flavonol)	20 μM/L	hnRNPA1	-	Pancreatic, thyroid cancer	CD18, K1 and 8505c, MDA-T85	*in vitro/in vivo*	↑ Apoptosis, ↓Cell proliferation	Pham et al. (2019)
	20–60 µM	↓DNMT1	NF-κB p65, p16^INK4α^	Esophageal cancer	9,706	*in vitro*	↑Apoptosis	Zheng et al. (2014)
		↓HDAC1						
	50 μM/L	↓HDAC1 ↓DNMT1 and ↓DNMT3a ↑mRNA	*DAPK1*, *BCL2L11*, *BAX*, *APAF1*, *BNIP3*, and *BNIP3L*	Leukemia	HL60 and U937	*in vitro/in vivo*	Cell cycle arrest	Alvarez et al. (2018)
							↑ Apoptosis	
							↑ Bax Activity	
							↑caspase Activity	
Kaempferol (Flavonol)	1.25–5 μM	↓DNMT1	*DACT2*	Colorectal Cancer	HCT116, HT29, and YB5	*in vitro/in vivo/in silico*	↑ Apoptosis	Lu et al. (2018)
							↓Proliferation	
							↓Migration	
							Wnt/β-catenin pathway	
	50–200 μM	↓HDAC2, 4, 7 or 8	-	Hepatoma and colon cancer	HepG2, Hep3B HCT-116	*in vitro/in silico*	↑ Apoptosis	Berger et al. (2013)
							↓ cell proliferation	
	50 μM	↓HDAC	p62 and *ATG5*	Gastric cancer	AGS, SNU-216, NCI-N87, SNU-638, and MKN-74	*in vitro*	↑Autophagy	Kim et al. (2018)
							↑Cell death	
							↓ Cell viability	
							↑caspase Activity	
							AMPKα/ULK1 and mTOR/p70S6K pathways	
Fisetin (Flavonol)	0–400 μM 160-mg/kg	histone demethylation	*RFXAP* and *KDM4A*	Pancreatic adenocarcinoma	BxPC-3, MiaPACA-2, PANC-1, and HPC-Y5	*in vitro/in vivo*	↑ Apoptosis	Ding et al. (2020)
							↓ Cell proliferation	
							DNA damage	
							Cell cycle arrest	
Apigenin (Flavone)	20–40 µM	↓ HDAC1 and HDAC3	p21	Prostate cancer	PC-3 and 22Rv1	*in vitro/in vivo*	↑ Apoptosis	Pandey et al. (2012)
		↑bax, ↓ bcl2					Cell cycle arrest	
	-	↓HDAC	*CDK1* and p21	Breast cancer	MDA-MB-231	*in vitro/in vivo*	↓Cell proliferation	Tseng et al. (2017)
		Induce histone acetylation					Cell cycle arrest	
Luteolin (Flavone)	0–30 μM	↓ AKT/mTOR-inducing H3K27Ac and H3K56Ac	*MMP9*	Breast cancer	Triple-negative breast cancer cells	*in vitro*	↓Cell proliferation,	Wu et al. (2021)
							↓Metastasis	
	-	↓ DNMT	*UHRF1* and p16	Colorectal cancer	-	*in vitro*	↑Cytotoxicity,	Krifa et al. (2014)
							Cell cycle perturbation	
Tangeretin derivative (Flavone)	14.6 μM	↓ DNMT 3B and HDACs 1, 2, and 4/5/9	p21	Prostate cancer	LNCaP	*in vitro*	↑ Apoptosis, upregulated Bad and Bax, downregulated Bcl-2, and activated caspase-3 and PARP	Wei et al. (2019)
Hesperetin (Flavanone)	-	↓histone H3K79 methylation,	*DOT1L*	Gastric cancer	-	*in vitro/in vivo*	↓Cell migration and ↓Invasion	Wang et al. (2021a)
Silibinin (Flavanone)	25–75 μg/ml	↑DNMT, ↓HDACs1-2	*EZH2*	Prostate cancer	DU145 and PC3	*in vitro*	↑ Apoptosis	Anestopoulos et al. (2016)
		↑H3K27me3						
Genistein (Isoflavone)	40 µM	DNA hypermethylation	*BRCA1*, *GSTP1*, *EPHB2,* and *BRCA2*	Prostate cancer	DU-145 and PC-3	*in vitro*	↓ Cancer cell growth	Adjakly et al. (2011)
	25–50 μM	↓ DNMT	*BTG3*	Prostate cancer	LNCaP, PC-3	*in vitro*	↑ Apoptosis	Majid et al. (2010)
		↑HAT						
		Histone modifications						
	2–20 μM/L	↓ DNMT	*RARB*, p16, and *MGMT*	Esophageal cancer	KYSE 510	*in vitro*	↓ Cancer cell growth	Fang et al. (2005)
	75 μM/L	↓ DNMT	*WNT5a*	Colon cancer	DLD-1, SW480, and SW1116	*in vitro*	↓ Cell proliferation	Wang and Chen, (2010)
Delphinidin (Anthocyanidin)	10–100 μM	↓ DNMT1, ↓ DNMT3a, ↓ I/II HDACs).	*Hmox1*, *Nqo1,* and Sod1	Skin cancer	JB6 P+	*in vitro*	↑Apoptosis	Kuo et al. (2019)
							↓Colony formation	
	50–150 mM	↓ HDAC3	p53	Prostate cancer	LNCaP	*in vitro*	↑Apoptosis	Jeong et al. (2016)
							↑ Caspase-3,7,8 activity	

### Cancer Prevention and Therapy by Epigenetically Active Flavonoids

#### Flavan-3-Ols/ Flavanols/ Catechins

Epigallocatechin gallate (EGCG) is a powerful polyphenolic, chemo-preventive compound isolated from green tea belongs to the catechin class of flavonoids (Singh et al., 2011). The other components of green tea consist of epigallocatechin, epicatechin-3-gallate and epicatechin. There are numerous *in vitro*, *in vivo*, and clinical studies that showed potential anti-proliferative, anti-angiogenic, pro-apoptotic and anti-invasive properties of EGCG (Singh et al., 2011). There are significant literatures demonstrating the impact of green tea components in involved in epigenetic modulations in cancer (Henning et al., 2013; Giudice et al., 2018; Jiang et al., 2021). Fang *et al.*, first time revealed that EGCG act as inhibitor of DNA hypermethylation of CpG islands by acting on DNMT (Fang et al., 2003). Another study has shown the profound effects of EGCG on human prostate cancer cells (Paschka et al., 1998). Green tea causes an accumulation of acetylated histone H3 in total cellular chromatin resulting in epigenetically reactivation of p21/waf1 and Bax in prostate cancer cells that leads to cell cycle arrest and apoptosis (Thakur et al., 2012).

Lee *et al.*, examined the regulation of androgen receptor acetylation by measuring histone acetyltransferase activity in androgen-dependent prostate cancer cells treated with green tea catechins (epicatechin, epigallocatechin and epigallocatechin-3-gallate). These catechins induce prostate cancer cell death, suppressed agonist-dependent androgen receptor (AR) activation and AR-regulated gene transcription (Lee et al., 2012). Another report showed that the combinatorial exposures of clofarabine and EGCG or genistein synergistically inhibited the growth of breast cancer cells (MCF7 and MDA-MB-231 cells) and induces apoptosis followed by *RARB* hypomethylation with consequent manifold increase in *RARB*, *CDKN1A,* and *PTEN* transcript levels. This combination promotes apoptosis and reactivates DNA methylation-silenced tumor suppressor genes in human breast cancer cells with unusual invasive prospective (Lubecka et al., 2018).

EGCG alters the expression of various tumor-suppressor genes by inhibiting DNA methyltransferases and histone deacetylases in human cervical cancer HeLa cells (Khan et al., 2015). Moreover, time-dependent exposure to EGCG resulted in reactivation of well-known tumor-suppressor genes (TSGs) in these cells due to marked transformations in the methylation of the promoter area of these genes (Khan et al., 2015). Recently Ciesielski et al., 2020 studied the impact of EGCG on the histone posttranslational modifications machinery along with chromatin remodeling in human endothelial cells (HMEC-1 and HUVECs origin). Results showed that EGCG increases methylation of both active (H3K4me3) and repressive (H3K9me3) chromatin marks and histone acetylation (H3K9/14ac, H3ac). These results indicated the broad epigenetic potential of EGCG concerning expression and action of epigenome modulators including HDAC5, and HDAC7, CREBP, KMT2A, p300 and LSD1 (Ciesielski et al., 2020).

Another study reported the anticancer mechanism of EGCG *via* synchronized transcriptional modification of numerous molecular targets through different signaling pathways in Hela cells (Kedhari Sundaram et al., 2020). In this study, transcriptional modulation of several epigenetic modifiers including histone modifiers and DNA methyltransferases (DNMT1, DNMT3A, DNMT3B, AURKA, AURKB, AURKC, PRMT6, PRMT7, KDM4A, KDM5C, HDAC5, HDAC6, HDAC7, HDAC11 and UBE2B) were observed. Downregulation of key signaling moieties of PI3K, Wnt and MAPK pathways, metastasis regulators, cell cycle regulators and pro-inflammatory moieties including CCNB1, CCNB2, TERT, PIK3C2B, PIK3CA, IL6, MMP2, MMP7 and MAPK8 were also detected (Kedhari Sundaram et al., 2020).

Kang et al., demonstrated that EGCG may hamper efficiently IR-induced injury to mouse normal hepatic cells (AML-12), and improve noticeably the radio-sensitivity of mouse hepatoma cells H22 to ^60^Coγ. They also revealed that EGCG played the key task of radio-sensitization on H22 cells because it activates the miR-34a/Sirt1/p53 signaling pathway (Kang et al., 2019). Deb *et al.*, reported that treatment of human prostate cancer cell lines (DUPro and LNCaP) with green tea polyphenols (GTPs) and their major constituent, epigallocatechin-3-gallate (EGCG) induced TIMP3 expression by epigenetic mechanisms (Deb et al., 2019). Furthermore, a clinical study on patients undergoing prostatectomy consuming EGCG showed an increase in plasma TIMP3 levels (Deb et al., 2019).

Dietary flavonoids have potential to modulate non-coding RNAs, including miRNAs in cancer (Ahmed et al., 2020; Yang et al., 2020; Singh et al., 2021). In a recent *in vivo* study by Kang et al., revealed that oral administration of EGCG suppresses miR483–3p induced metastasis of hepatocellular carcinoma (Kang et al., 2021). EGCG modulate non-coding RNAs and inhibit tumor growth by targeting LINC00511/miR-29b/KDM2A axis in gastric cancer (Zhao et al., 2020). EGCG-capped gold nanoparticle significantly increased expression of tumor suppressor let-7a and miR-34a miRNAs (Mostafa et al., 2020).

#### Flavonols

Flavonols (3-hydroxylavones) are most prevalent flavonoids in food. Quercetin, myricetin, kaempferol and fisetin are the most common plant flavonols found in many vegetables and fruits, e.g., onions, apples, strawberries etc. The defensive impacts of quercetin on human health are facilitated by multidimensional, pleiotropic action still from an epigenetic perspective (Russo and Ungaro, 2019).

Quercetin modulates the expression of various chromatin modifiers and declines the activity of HDACs, DNMTs and HMTs in a dose-dependent manner in human cervical cancer (HeLa) cells (Kedhari Sundaram et al., 2019). It also downregulated global DNA methylation concentrations in a dose- and time-dependent manner and tested tumor suppressor genes showed steep dose-dependent decrease in promoter methylation with the restoration of their expression. Quercetin along with BET inhibitors promoted apoptosis and decreases the cell proliferation and sphere-forming ability by pancreatic cancer cells. It was also evidenced that quercetin also mediates some anti-tumor effects with the help of hnRNPA1 which is a nuclear protein well-known to monitor mRNA export and mRNA translation of anti-apoptotic proteins (Pham et al., 2019). Quercetin also induced let-7c which decreased pancreatic tumor growth by posttranscriptional activation of Numbl and indirect inhibition of Notch (Nwaeburu et al., 2016); Zheng et al. (2014) reported that nanoliposomal quercetin combined with butyrate modulated aberrant epigenetic alteration in Eca9706 cells *via* epigenetic-NF-κB signaling. In this study reverse expressions of global DNMT1, HDAC1, NF-κB p65 and Cyclin D1 were down-regulated, although expressions of p16INK4α and caspase-3 were up-regulated. Furthermore, quercetin modulate miR-1-3p/TAGLN2 (Wang et al., 2021b), miR-197/IGFBP5 (Hu et al., 2020), miR-16–5p/WEE1 (Wang et al., 2020a), miR-22/WNT1/β-catenin (Zhang et al., 2019a), miR-16/HOXA10 (Zhao et al., 2019), TP53/miR-15/miR-16 (Ahmed Youness et al., 2018) axes as well as miR15a/16 (Ramos et al., 2021), miR-200b-3p (Nwaeburu et al., 2017), miR-145 (Zhou et al., 2015), and miR-146a (Tao et al., 2015) in various cancers.

Kaempferol (3,4′,5,7-tetrahydroxyflavone) is a potential HDAC inhibitor and an anti-cancer agent against many types of cancers (Imran et al., 2019). Berger *et al.*, reported first time that kaempferol has a distinctive epigenetic activity by inhibition of HDACs (Berger et al., 2013). The *in-silico* docking analysis fits kaempferol into binding pocket of HDAC2, 4, 7 or 8 and *in vitro* profiling of all conserved human HDACs of class I, II and IV demonstrated that it inhibited all tested HDACs. Furthermore, kaempferol stimulates hyperacetylation of histone H3 in HepG2 and Hep3B (hepatoma cancer cell lines) as well as on HCT-116 (colon cancer cell line) (Berger et al., 2013). Kaempferol induces autophagic cell death *via* IRE1-JNK-CHOP signaling and inhibiting HDAC/G9a axis in gastric cancer cells (Kim et al., 2018). In lung cancer A549 cell, kaempferol induces miR-340 expression (Han et al., 2018) which is known to induce apoptosis and inhibit cell proliferation in NSCLC (Fernandez et al., 2014).

Flavonol, fisetin is also a powerful anticancer agent, used to inhibit different stages of cancer cells, induce apoptosis, inhibit cell growth, prevent cell cycle progression, cause PARP cleavage, and modulate the expressions of Bcl-2 family proteins in various cancer cell lines (Imran et al., 2020). It also suppresses the activation of the ROS/ PKCα/ p38 MAPK and ERK1/2 signaling pathways, down-regulates the level of the oncoprotein securin and lowers the NF-κB activation (Pal et al., 2016). Recently, Ding *et al.*, revealed that fisetin inhibits proliferation of pancreatic adenocarcinoma by inducing DNA damage *via* RFXAP/KDM4A-dependent histone H3K36 demethylation (Ding et al., 2020).

#### Flavones

Flavones are a group of flavonoids that contain the backbone of 2-phenylchromen-4-one (2-phenyl-1-benzopyran-4-one) having diverse pharmacological properties and are commonly found in herbs such as celery, parsley and in almost all edible cereal species. Apigenin, luteolin, tangeretin, chrysin, Tricin, baicalein, rhoifolin and 6-hydroxyflavone are some common flavones (Barreca et al., 2020).

Pandey et al. (2012) reported first time that apigenin inhibits class I HDACs, particularly HDAC1 and HDAC3, alters chromatin to induce growth arrest and apoptosis in human prostate cancer cells. Apigenin inhibited MDA-MB-231 breast cancer cell proliferation and tumor growth by induction of G2/M arrest and histone H3 acetylation-mediated p21 expression (Tseng et al., 2017). Apigenin enhances miR-16 (Chen et al., 2016) and miRNA215–5p (Cheng et al., 2021) expression to inhibits glioma and colon cancer growth respectively, as well as chemo-sensitize doxorubicin-resistant liver cancer cells by targeting miR-520b/ATG7 axis (Gao et al., 2018).

Recently Wu et al. (2021) discovered that luteolin inhibited the proliferation and metastasis of androgen receptor-positive triple-negative breast cancer cell by epigenetic regulation of MMP9 expression through a reduction in the levels of AKT/mTOR-inducing H3K56Ac and H3K27Ac. Earlier it was also revealed that luteolin suppresses the metastasis of triple-negative breast cancer, downregulates the *ß*-catenin expression for reversing epithelial-to-mesenchymal transition (Lin et al., 2017). In colorectal cancer cells Luteolin induces apoptosis by the downregulations of UHRF1, calpain, and DNMT1 expressions. This research further suggests that calpain might be involved in the epigenetic code inheritance by regulating the epigenetic integrator UHRF1(Krifa et al., 2014). In human prostate cancer (PC-3) cells, Luteolin and gefitinib regulate cell cycle pathway genes (*CCNA2*, *CCNE2*, *CDC25A*, *CDKN1B*, and *PLK*-1) through a mutual mechanism involving EGFR-associated tyrosine kinase (Markaverich et al., 2010). Authors suggested that these phytochemicals likely modulate the epigenetic control of gene expression as previously shown by their group that luteolin interacts with type II binding sites on histone H4 (Shoulars et al., 2010). Recently, Farooqi et al. and Mishan et al. have systematically reviewed the potent ability of luteolin to modulate miRNA expression in various cancers (Farooqi et al., 2020; Mishan et al., 2021). A derivative of tangeretin prevents the progress of human prostate cancer cells by epigenetically restoring p21 gene expression and inhibits cancer stem-like cell proliferation (Wei et al., 2019).

Baicalein (5,6,7-trihydroxyflavone) suppresses cancer cell proliferation, cell cycle arrest and induces apoptosis in human prostate, breast, T24 bladder and myeloma cancer cells (Gao et al., 2016). Lai *et al.*, studied the epigenetic role of baicalin hydrate in nasopharyngeal carcinoma (NPC) and identified that it inhibits NPC cell growth both *in vivo* and *in vitro*. Furthermore, instead of DNA methylation, baicalin hydrate increased of m6A RNA methylation and promoted Suv39H1 gene splicing. (Lai et al., 2018). It is also documented that baicalin improves the developmental proficiency of *in vitro* cultured mouse embryos through reticence of cellular apoptosis and HSP70 expression and enhancement of DNA methylation (Qi et al., 2016). Several recent studies have shown that baicalein modulate the expression of miR-183 (Lei et al., 2021), miR-139–3p and miR-196b-5p (Ma et al., 2021), and miR-25 (Örenlil Yaylagül and Ülger, 2020) in various cancers.

#### Flavanones

Flavanones are aromatic, colourless ketones mainly present in citrus fruits (Barreca et al., 2017). Hesperetin, isosakuranetin, naringin, naringenin, isosakuranetin and eriodictyol and their particular glycosides are some main flavanones (Khan et al., 2013). Hesperetin is a common citrus flavanone that endorses DOT1L degradation and decreases histone H3K79 methylation to prevent gastric cancer metastasis, showing its epigenetic effect (Wang et al., 2021a). Natural flavonolignan, silibinin is the most effective phytochemical of silymarin. It is active both alone and in combination with other chemotherapeutic and epigenetic agents, substantially inhibit the growth of different cancer cells. It synergizes with DNA methyltransferase and histone deacetylase inhibitors in upregulating e-cadherin expression, also inhibits the invasion and migration of human non-small cell lung cancer cells. These results are highly substantial since failure of E-cadherin and metastatic dispersed of the illness *via* epithelial-to-mesenchymal transition is associated with poor prognosis and high mortalities in this type of cancer cells (Mateen et al., 2013). In human prostate cancer (DU145 and PC3) cells, silibinin reduces gene expression levels of EZH2 accompanied by an increase in H3K27me3 levels (Anestopoulos et al., 2016). Such responses were dependent on decreased expression levels of phosphorylated EZH2 (ser21) and phosphorylated Akt (ser473). Moreover, it also exerted other epigenetic impacts involving, decrease histone deacetylases 1–2 (HDACs1-2) expression levels while it increases total DNA methyltransferase (DNMT) activity, proving that it induces epigenetic alterations in human prostate cancer cells. (Anestopoulos et al., 2016). Hossainzadeh et al. reported that silibinin encapsulated in polymersome nanoparticles supress the expression of oncogenic miRNAs miR-125b and miR-182 (Hossainzadeh et al., 2019).

Naringenin (4,5,7 trihydroxyflavanone) is an aglycone form of naringin found in citrus fruits. When it combined with suberoylanilide hydroxamic acid (HDAC inhibitor), synergistically improved transamidation activity and suberoylanilide hydroxamic acid induced cytotoxicity in neuroblastoma cells which showed no cytotoxicity on normal non-malignant cells (Ling et al., 2012). This suggest that naringenin possesses effective histone deacetylase inhibitory activity; however, more comprehensive studies are needed understand its epigenetic potential.

#### Isoflavones

Isoflavones are naturally occurring isoflavonoids, mainly found in legumes, soy beans, and soy foods. They have several potent pharmacological activities like anti-inflammatory, antioxidant, antimicrobial, and anticancer (Panche et al., 2016). It is also well evidential that they exert estrogenic and/or antiestrogenic impacts. Isoflavones are considered as chemoprotective in nature and used in various type of alternative therapies for a broad range of hormonal ailments including several types of cancers, osteoporosis, menopausal problems and cardiovascular diseases (Taku et al., 2011; Vitale et al., 2012; Qin et al., 2013; Sathyapalan et al., 2018; Nakai et al., 2020). There are conflicting reports that isoflavones disrupt endocrine function; however, it appears the most common harmful effect is mild and appears at the gastrointestinal level (Křížová et al., 2019; Gómez-Zorita et al., 2020). Some common examples of isoflavones are Daidzein, Genistein, Genistin and Glycitein.

Among all isoflavones, genistein is the most potent and biologically active phytochemical, demonstrating different *in vivo* and *in vitro* anticancer and anti-proliferative effects on numerous types of human cancers (Spagnuolo et al., 2015). Prostate cell lines (DU-145 and PC-3) when treated with soy phytoestrogens, genistein and daidzein, cause decrease in DNA methylation at *EPHB2*, *BRCA1* and *GSTP1* promoters (Adjakly et al., 2011). Karsli-Ceppioglu *et al.*, reported that phytoestrogens (genistein and daidzein) modulate genome-wide DNA methylation status in prostate cancer. They found that methylation profiles of 58 genes have been modified by genistein and daidzein treatments in prostate cancer DU-145 and LNCaP cell lines (Karsli-Ceppioglu et al., 2015). Notably, the methylation frequencies of the *hTERT*, *MAD1L1*, *KDM4B* and *TRAF7* genes were remarkably altered by genistein treatment. Genistein regulates miRNAs expression in pan cancer (Javed et al., 2021). In head and neck cancer, genistein activate miR-34a/RTCB axis that results in ROS-associated apoptosis, decrease in stemness properties, and inhibition of EMT (Hsieh et al., 2020). Recently, Imai-Sumida *et al.*, reported that genistein suppress kidney cancer by repressing HOTAIR/chromatin remodeling pathways (Imai-Sumida et al., 2020). Conversely, Allred *et al.,* reported that dietary genistin as well as genistein can stimulates estrogen-dependent breast cancer cell growth *in vivo* (Allred et al., 2001). These conflicting reports compel a need for in-depth mechanistic studies to understand the biological effects of these dietary compounds, including their epigenetic modifying potentials, cytotoxicity, and anticancer properties.

#### Anthocyanidins

Anthocyanidins are the sugar-free counterparts of anthocyanins and are highly important water-soluble pigments found in plants. Among all anthocyanidins, delphinidin is the most potent and abundant flavonoid found in pigmented fruits (especially blueberry) and vegetables (Khoo et al., 2017).

Kuo *et al.*, reported that delphinidin epigenetically re-activates Nrf2-ARE pathway and prevents neoplastic transformation of mouse skin JB6 P+ cells (Kuo et al., 2019). The Nrf2-ARE pathway activation was associated with demethylation of 15 CpG sites in the mouse Nrf2 promoter region between nucleotide -1,226 and -863 from the transcription start site. The decreased CpG methylation proportion in the Nrf2 promoter region was consistent with detected declines in the protein expression of DNA methyltransferases 1 (DNMT1), DNMT3a, and class I/II histone deacetylases (HDACs) (Kuo et al., 2019). Jeong *et al.*, identified the epigenetic modulators that mediate the apoptotic effect of delphinidin (major anthocyanidin compound) in human prostate cancer cells (Jeong et al., 2016). They treated these cancer cells with delphinidin and observed an increase in caspase-3, -7, and -8 activity along with an increased histone deacetylase activity. Amongst all class I HDACs, the activity of HDAC3 was exclusively prevented by delphinidin. Moreover, the apoptosis induced by delphinidin was reliant on caspase-mediated cleavage of HDAC3, resulting in the stabilization and acetylation of p53. They also observed that this anthocyanidin effectively upregulated pro-apoptotic genes that are definitely regulated by p53 and also downregulated numerous anti-apoptotic genes (Jeong et al., 2016). Delphinidin targets HOTAIR/miR-34a axis to suppress the breast carcinogenesis (Han et al., 2019) and inhibits colorectal cancer metastasis by inducing miR-204–3p expression (Huang et al., 2019).

## Conclusion

The existing literatures on cancer pathobiology has established that genetics and epigenetics play a central role in cancer initiation and progression. The reversible nature of epigenetic changes can be exploit for a better therapeutic intervention. Epigenetic modifiers such as DNMTs, HATs, HMTs, HDACs, and others can be modulated or inhibited by naturally occurring substances such as phytochemicals (Figure 2). In this review, several flavonoids (e.g., flavones, flavanones, flavonols) were described along with their epigenetic modulation, therapeutic, and chemo-preventive potentials in cancer (Table 1). Various plant-based drugs, including vinca alkaloids (e.g., vinblastine and vincristine), taxanes (e.g., paclitaxel and docetaxel), camptothecins, and others are routinely used for cancer treatment. There are numerous phytochemicals, including flavonoids with potent anticancer activity are in various preclinical and clinical trial stages. However, there are some limitations, including their low water solubility, poor bioavailability, rapid uptake by normal cells, poor therapeutic index and adverse effects on liver. Developing novel strategies such as nanocarriers can overcome these drawbacks.

The emerging role of phytochemicals in cancer therapy and prevention is enormous. Their potential to rectify epigenetic alterations of cancer cells add substantial armaments in fight against cancer. Combinatorial therapies using plant-based epigenetic modifiers with existing chemo and targeted therapies can help manage the disease better and reduce the side effects. However, an extensive research is needed into identification and characterization of anticarcinogenic phytochemicals and their respective mechanisms of actions. Given the fast pace of technological developments, this seems to be a promising pursuit in our battle against cancer.

## Glossary

ABCG2: ATP-binding cassette G2
AKT: Ak strain transforming
AMPK: 5' adenosine monophosphate-activated protein kinase
APAF1: Apoptotic protease activating factor 1
AR: Androgen receptor
ARE: Antioxidant responsive element
ATG5 /7: Autophagy related 5 /7
AURKA /B /C: Aurora kinase A /B /C
BAX: BCL2 associated X
BCL-2: B-cell lymphoma 2
BCL2L11: Bcl-2-like protein 11
BDC: Bile duct cancer
BET: Bromodomain and extraterminal
BNIP3: BCL2 and adenovirus E1B 19-kDa-interacting protein 3
BNIP3L: BCL2 and adenovirus E1B 19-kDa-interacting protein 3-like
BRCA1 /2: Breast cancer gene 1 /2
BTG3: B-cell translocation gene 3
C/EBPα: CCAAT enhancer-binding protein alpha
CAF: Chromatin assembly factor
CCNA2 /B1 /B2 / D1 /E1 /E2: Cyclin A2 /B1 /B2 /D1 /E1 /E2
CD: Cluster of differentiation
CDC25A: Cell division cycle 25 A
CDH1 /13: Cadherin 1 /13
CDK1: Cyclin-dependent kinase 1
CDKN1A /1B: Cyclin dependent kinase inhibitor 1A /1B
CHOP: C/EBP homologous protein
CREB: cAMP-response element binding protein
DACT2: Disheveled-associated antagonist of β-catenin homolog 2
DAPK1: Death-associated protein kinase 1
DOT1L: DOT1-like histone H3K79 methyltransferase
DR5: Death receptor 5
E2F1: E2F transcription factor 1
EED: Embryonic ectoderm development
EGFR: Epidermal growth factor receptor
EMT: epithelial-mesenchymal transition
EPHB2: EPH receptor B2
ERK: Extracellular signal-regulated protein kinase
EZH2: Enhancer of zeste homolog 2
GAS1: Growth arrest specific 1
GBC: Gall bladder carcinoma
GSTP1: Glutathione S-transferase pi 1
hMLH1: Human mutL homolog 1
HMOX1: Heme oxygenase 1
hnRNPA1: Heterogeneous nuclear ribonucleoprotein A1
HOTAIR: HOX transcript antisense RNA
HOXA10: Homeobox A10
HSP70: Heat shock proteins 70 kda;
hTERT: Human telomerase reverse transcriptase
ICAM1: Intercellular adhesion molecule 1
IGFBP5: Insulin like growth factor binding protein 5
IL: Interleukin
IRE1: Inositol-requiring enzyme1
JNK: c-Jun N-terminal kinase
KDM4A /4B /5C: Lysine (K)-specific demethylase 4A /4B /5C
KMT2A: Lysine (K)-specific methyltransferase 2A
Let -7: Lethal-7
LSD1: Lysine-specific demethylase 1
m6A: N⁶-methyl-adenosine
MAD1L1: MAD1 mitotic arrest deficient 1-like 1
MALAT1: Metastasis associated lung adenocarcinoma transcript 1
MAPK: Mitogen-activated protein kinase
MEG3: Maternally expressed gene 3
MGMT: O6-methylguanine-DNA methyltransferase
MMP: Matrix metallopeptidase
mTOR: Mammalian target of rapamycin
NF-κB: Nuclear factor kappa B
NQO1: NAD(P)H quinone dehydrogenase 1
NRF2: Nuclear factor erythroid 2-related factor 2
NSCLC: Non-small cell lung cancer
NUMBL: NUMB Like Endocytic Adaptor Protein
p70S6K: Ribosomal protein S6 kinase beta-1
PARP: Poly (ADP-ribose) polymerase
PI3K: Phosphoinositide 3-kinases
PIK3C2B: Phosphatidylinositol-4-phosphate 3-kinase catalytic subunit type 2 beta
PIK3CA: Phosphatidylinositol-4,5-bisphosphate 3-kinase catalytic subunit alpha
PKCα: Protein kinase C alpha
PLK-1: Polo-like kinase 1
PRC: Polycomb repressive complex
PRDM2: PR domain zinc finger protein 2
PRMT6 /7: Protein arginine N-methyltransferase 6 /7
PTEN: Phosphatase and tensin homolog
PTENP1: PTEN pseudogene 1
RARB: Retinoic acid receptor beta
RFXAP: Regulatory factor X associated protein
ROS: Reactive oxygen species
RTCB: RNA 2',3'-cyclic phosphate and 5'-OH ligase
RUNX3: Runt-related transcription factor 3
S100P: S100 calcium binding protein P
SCUBE2: Signal peptide-CUB-EGF domain-containing protein 2
SOD1: Superoxide dismutase 1
SUV39H1: Suppressor of variegation 3-9 homolog 1
SUZ12: Suppressor of zeste homolog 12
TAGLN2: Transgelin 2
TIMP3: Tissue inhibitor of metalloproteinase 3
TRAF7: TNF receptor associated factor 7
UBE2B: Ubiquitin conjugating enzyme E2 B
UHRF1: Ubiquitin like with PHD and ring finger domains 1
ULK1: Unc-51 like autophagy activating kinase
WEE1: WEE1 G2 Checkpoint Kinase
WISP2: WNT1-inducible-signaling pathway protein 2
WNT: Wingless/Integrated
WNT5a: Wnt family member 5A
γ-H2AX: Gamma H2A histone family member X
